# The associations of physical activity and sedentary behavior with self-rated health in Chinese children and adolescents

**DOI:** 10.1371/journal.pone.0304693

**Published:** 2024-05-31

**Authors:** Yahan Liang, Youzhi Ke, Yang Liu

**Affiliations:** 1 School of Physical Education, Shanghai University of Sport, Shanghai, China; 2 Department of Human Movement Studies and Special Education, Darden College of Education & Professional Studies, Old Dominion University, Norfolk, VA, United States of America; 3 Shanghai Research Center for Physical Fitness and Health of Children and Adolescents, Shanghai University of Sport, Shanghai, China; Universiti Malaya, MALAYSIA

## Abstract

**Objective:**

The study aimed to analyze the independent and joint associations of physical activity (PA) and sedentary behavior (SB) with self-rated health (SRH) among Chinese children and adolescents.

**Methods:**

Cross-sectional data on moderate-to-vigorous physical activity (MVPA), school-based PA, extracurricular physical activity (EPA), screen time (ST), homework time, and SRH were assessed through a self-report questionnaire in the sample of 4227 Chinese children and adolescents aged 13.04 ± 2.62 years. Binary logistic regression was used to compare gender differences in PA, SB, and SRH among children and adolescents, and analyses were adjusted for age and ethnicity.

**Results:**

In independent associations, boys and girls engaging in ≥60 min/day of MVPA and >1 hour/day of EPA reported optimal SRH. Only boys who participated in >1 hour/day of school-based PA were significantly more likely to have optimal SRH (OR = 1.49, 95%CI = 1.19–1.86). Only girls who had ≤2 hours/day of ST were significantly associated with optimal SRH (weekdays: OR = 1.38, 95%CI = 1.10–1.74; weekends: OR = 1.40, 95%CI = 1.14–1.71; whole week: OR = 1.42, 95%CI = 1.16–1.73). In joint associations, regardless of SB recommendation, meeting PA recommendation was significantly associated with optimal SRH in both boys (meet PA and SB recommendations, OR = 1.61, 95%CI = 1.03–2.50; meet PA but not SB recommendations, OR = 2.40, 95%CI = 1.57–3.65) and girls (meet PA and SB recommendations, OR = 3.72, 95%CI = 2.08–6.65; meet PA but not SB recommendation, OR = 4.27, 95%CI = 2.09–8.75).

**Conclusion:**

Increased PA and reduced SB were positively associated with optimal SRH in Chinese children and adolescents. Notably, lower ST positively influenced only girls’ SRH. Meeting PA recommendation is more impactful than meeting SB recommendation for improving SRH in Chinese children and adolescents. Future studies could explore these associations using objective measures of PA and SB in China.

## 1. Introduction

In China, the detection rate of depression among adolescents was 24.6% from 2019 to 2020 [1]. The National Report on Nutrition and Chronic Diseases in Chinese Residents in 2020 reported that the prevalence of overweight and obesity rates among Chinese children and adolescents aged 6 to 17 were up to 19% [2]. Childhood and adolescence are critical periods for physical growth and psychological development [3, 4], which provide a valuable window for identifying risk facts and preventing health problems in later life [4, 5]. Research showed that managing self-rated health (SRH) early in life may be a principal strategy to prevent morbidity in adulthood [6]. SRH reflects individuals’ subjective perception of their overall health status [7]. SRH status is consistent with objective health status [8, 9], and it has been identified as an independent predictor of morbidity and mortality [10, 11]. SRH is commonly used in large population surveys around the world as a measure of people’s health [12] and has been confirmed to be a stable measure of health status from adolescence to early adulthood [6, 13], serving as a valid indicator of physical and mental health in adolescents [6, 13–15]. Consequently, comprehending the factors associated with SRH in Chinese children and adolescents is of paramount importance.

SRH is a multifactorial composite associated with a wide range of medical, psychological, social, and lifestyle factors among adolescents [16]. Previous evidence has revealed several factors associated with SRH in children and adolescents, such as mental health (e.g., loneliness, hopelessness, and depression) [16–18], physical health (e.g., overweight, headache, and stomachache) [18, 19], family environment (e.g., family economic status, quarrels in the family, and unemployed parents) [18, 20, 21], social development (e.g., self-esteem, social support, and competence) [22, 23], and health-related behaviors (e.g., dietary habits, drinking, smoking, physical activity (PA), and sedentary behavior (SB)) [18, 20, 24–27]. In the realm of children and adolescent health, PA and SB occupy pivotal roles, exerting substantial impact [28, 29]. The Canadian 24-Hour Movement Guidelines recommended that children and adolescents aged 5–17 years should do at least 60 min/day of moderate-to-vigorous physical activity (MVPA) and not more than 2 hours/day of screen time (ST) [30]. Additionally, the Ministry of Education of the People’s Republic of China announced a policy requiring Chinese provincial and municipal education departments to ensure that students engage in one hour of PA at school [31]. Worldwide, studies have embarked on exploring the intricate associations between PA, SB, and SRH in the domain of children and adolescents [32].

Existing research on the independent associations of PA and SB with SRH among children and adolescents has generated a diverse array of findings. Many studies have shown that meeting the PA recommendations (≥60 min/day of MVPA) was positively associated with good SRH among adolescents in European countries such as Poland, America, and Portugal [33–36]. Curtin et al. showed that engaging at least once a week in school-based organized sports was not significantly associated with good SRH in Korean adolescents [37]. Besides, adolescents who did not meet the SB recommendations (≤2 hours/day of ST) were about 30% more likely to report poor SRH in Canada [25], which disagrees with the result [27]. Spanish boys who used computers most on weekdays were more likely to consider their health level as low, but not girls [38].

Findings on the joint associations of PA and SB with SRH among children and adolescents are scarce. Matin et al. found that in their study, children and adolescents with ≤2 hours/day of ST—>4 hours/week of PA combination had the highest odds ratio (OR) for good SRH in Iran [39]. Tebar et al. showed that adolescents who participated in ≥4 hours of SB per day were associated with poor SRH regardless of whether they participated in ≥300 minutes of PA per week in Brazil [40]. Furthermore, a study found that Canadian adolescents who met both the PA and SB recommendations were significantly more likely to have good SRH [41]. Overall, these findings were inconsistent and varied by gender and period (weekday and weekend) [38, 42, 43].

The perception of health status may vary significantly across countries, as cultural and environmental factors play a substantial role in shaping this perception [35]. As a result, the associations of PA and SB with SRH may differ by country. However, research investigating these associations among adolescents in China is limited [21, 44]. No studies have examined the associations of PA and SB with SRH among children and adolescents in China. Addressing these critical knowledge gaps, the study aimed to examine the independent and joint associations of PA (MVPA, school-based PA, extracurricular physical activity (EPA)) and SB (screen time, homework) with SRH among Chinese children and adolescents of different genders and periods (weekdays and weekends).

## 2. Methods

### 2.1 Study design and participants

This study adopted a cross-sectional study design to analyze the associations of PA and SB with SRH in Chinese children and adolescents. Data collection occurred during the period spanning September 1 to December 31, 2019. The study participants were drawn from a diverse set of 34 elementary, middle, and high schools situated in the expansive Yangtze River delta region of Eastern China. This region encompasses approximately 358,000 square kilometers and was inhabited by an estimated 227 million residents in 2019, spanning across Jiangsu, Anhui, and Zhejiang provinces, as well as the Shanghai municipality. The study adhered to specific inclusion criteria, which encompassed three key dimensions: 1) voluntary willingness to participate, 2) students capable of comprehending and completing the questionnaire, and 3) students enrolled in grades 3 through 12. Students below the third grade were excluded due to potential limitations in their ability to comprehend the questionnaire. Sample selection employed a multistage sampling approach. In the initial stage, a convenience sampling method was utilized to select schools within the target regions. Subsequently, in the second stage, the random cluster sampling method was employed to select classes within the target grade levels within the selected schools. All students in the chosen classrooms were invited to partake in the survey. To ensure the quality of the study, trained researchers gave the participants detailed instructions on how to complete the questionnaire before the survey, and the researchers explained questions that were unclear to the participants when they took the questionnaire. The survey garnered a total of 4,536 student participants. Following a rigorous data cleaning process, which involved the removal of missing and anomalous values from all independent and dependent variables, the final analysis was conducted on a dataset comprising 4,227 children and adolescents.

The study was approved by the Institutional Review Board (IRB) of the Shanghai University of Sport (SUS) and authorized by the participants’ schools and teachers. The IRB of SUS approved that the verbal consent was credible and adequate to conduct this study. Participation in the survey was voluntary, and verbal informed consent was obtained from participants and their legal guardians before data collection. The entire process was witnessed by the participant’s teacher.

### 2.2. Measures

MVPA, SB, ST, and homework were assessed using the items from the Health Behavior in School-aged Children (HBSC) survey questionnaire, which has proven reliable in Chinese children and adolescents [45]. These items have been widely used in studies of Chinese children and adolescent populations [46, 47]. Considering that the school-based PA is more likely to take place on weekdays and EPA is more likely to be carried out on weekends among Chinese school-aged children, it is unnecessary to examine the associations between these two indicators and SRH at different periods (weekdays and weekends). Hence, this study only analyzed the school-based PA and EPA during the whole week.

#### 2.2.1. Independent variables: PA and SB

The participants were asked how many days they engaged in at least 60 minutes of MVPA on weekdays and weekends, respectively. The answers were given on a 6-point scale (0 to 5 days) for weekdays, then dichotomized to 5 days/week and <5 days/week, and on a 3-point scale (0 to 2 days) for weekends, then divided into 2 days/week and <2 days/weeks. The two responses add up to the number of days in the whole week. Responses were dichotomized into two categories: 7 days/weeks (meeting the PA recommendations) and <7 days/weeks (not meeting the PA recommendations) [30]. School-based PA and EPA were examined using the following questions, respectively: “How many hours a day did you spend in school physical activities? (including physical education, the class-break exercise or morning exercises, and extracurricular activities in school)”, “How many hours a day do you usually take physical exercise outside school class so that you get out of breath or sweat?” The answers were given on a 4-point scale (1 = never, 2 = less than 30 minutes, 3 = 30 minutes to an hour, 4 = more than an hour). The responses of school-based PA were dichotomized into two categories: >1 hour/day (4) and ≤1 hour/day (1–3). The responses of EPA were categorized the same as the school-based PA.

ST was measured by asking participants how many hours per day they watched television and used mobile phones/iPads and computers in their leisure time on weekdays and weekends, respectively. Homework was assessed by asking participants how many hours of homework they did each day in their spare time on weekdays and weekends, respectively. The answers were given on a 5-point scale (1 = never, 2 = half-hour, 3 = one hour, 4 = two hours, 5 = at least 3 hours), adding up ST and homework hours were SB hours. We dichotomized ST into two categories: ≤2 hours/day (meeting the SB recommendations) and >2 hours/day (not meeting the SB recommendations) [30]. The responses for SB and homework were categorized the same as for ST.

#### 2.2.2. Outcomes: Self-rated health

SRH was measured via the question: ‘‘How would you describe your health?” options included: 1 = very good, 2 = good, 3 = fair, 4 = poor, and 5 = very poor. The responses were categorized into optimal SRH (1–2) versus other SRH (3–5). SRH is a stable predictor during adolescence and showed good reliability in measuring general health among Chinese youth [6, 13, 48].

### 2.3. Statistical analysis

The normality of the continuous variables (i.e. Age) was analyzed with the Kolmogorov–Smirnov test. Due to the non-normal distribution observed for continuous variables (*p* <0.001), data were expressed as mean ± standard deviation (M ± SD) of continuous variables and frequency and percentage of categorical variables. The statistical differences between boys and girls were tested with the Mann-Whitney test for continuous variables and the chi-square test for categorical variables. The odds of optimal SRH according to PA and SB categories were analyzed by binary logistic regression. All analyses were carried out separately for boys and girls and controlled for age and ethnicity. Analyses were conducted using IBM SPSS V.26, with a significance level of *p* <0.05.

## 3. Results

Table 1 shows the demographic characteristics of the participants. The present study sample included 4227 children and adolescents: 2024 boys (47.88%) and 2203 girls (52.12%). The mean age was 13.04 ± 2.62, ranging between 8 and 17 years. Table 2 shows the descriptive statistics of PA, SB, and SRH of the participants. There were significant differences in MVPA (*p* < 0.001), school-based PA (*p* < 0.001), EPA (*p* < 0.001), SB on weekdays (*p* = 0.005), ST on weekdays (*p* < 0.001), PA and SB combination (*p* < 0.001), and SRH (*p* < 0.001) between boys and girls. In short, Boys had more MVPA, school-based PA, EPA, SB on weekdays, ST on weekdays, and were more prone to report optimal SRH than girls. In addition, 12.04% of children and adolescents met the PA guidelines recommendations, and 39.44% met the SB guidelines recommendations in the Yangtze River Delta.

**Table 1:** Demographics of the study participants.

Variables		All (n = 4227)	Boys (n = 2024)	Girls (n = 2203)	*P*
Age (years), M±SD		13.04±2.62	12.81±2.58	13.26±2.63	<0.001
Ethnicity, n (%)					0.001
	Han	4145 (98.06)	1999 (98.76)	2146 (97.41)	
	Minority	82 (1.94)	25 (1.24)	57 (2.59)	
Area, n (%)					<0.001
	Anhui	685 (16.21)	293 (14.48)	392 (17.79)	
	Jiangsu	671 (15.87)	349 (17.24)	322 (14.62)	
	Shanghai	2682 (63.45)	1355 (66.95)	1327 (60.24)	
	Zhejiang	189 (4.47)	27 (1.33)	162 (7.35)	
School grade, n (%)					<0.001
	3-6th	1213 (28.70)	642 (31.72)	571 (25.92)	
	7-9th	2030 (48.02)	1022 (50.49)	1008 (45.76)	
	10-12th	984 (23.28)	360 (17.79)	624 (28.33)	

M±SD, mean ± standard deviation.

**Table 2:** Descriptive statistics of physical activity, sedentary behavior, and self-rated health.

Variables			All (n = 4227)	Boys (n = 2024)	Girls (n = 2203)	*P*
PA						
	MVPA, n (%)					
		Weekdays				<0.001
		5 days/week	1013 (23.96)	570 (28.16)	443 (20.11)	
		<5 days/week	3214 (76.04)	1454 (71.84)	1760 (79.89)	
		Weekends				<0.001
		2 days/week	1156 (27.35)	655 (32.36)	501 (22.74)	
		<2 days/week	3071 (72.65)	1369 (67.64)	1702 (77.26)	
		Whole week				<0.001
		7 days/week	509 (12.04)	313 (15.46)	196 (8.90)	
		<7 days/week	3718 (87.96)	1711 (84.54)	2007 (91.10)	
	School-based PA					<0.001
		>1 hour/day	1089 (25.76)	607 (29.99)	482 (21.88)	
		≤1 hour/day	3138 (74.24)	1417 (70.01)	1721 (78.12)	
	EPA					<0.001
		>1 hour/day	772 (18.26)	470 (23.22)	302 (13.71)	
		≤1 hour/day	3455 (81.74)	1554 (76.78)	1901 (86.29)	
SB						
	SB, n (%)					
		Weekdays				0.005
		≤2 hours/day	1372 (32.46)	614 (30.34)	758 (34.41)	
		>2 hours/day	2855 (67.54)	1410 (69.66)	1445 (65.59)	
		Weekends				0.640
		≤2 hours/day	489 (11.57)	239 (11.81)	250 (11.35)	
		>2 hours/day	3738 (88.43)	1785 (88.19)	1953 (88.65)	
		Whole week				0.731
		≤2 hours/day	357 (8.45)	174 (8.60)	183 (8.31)	
		>2 hours/day	3869 (91.55)	1849 (91.40)	2020 (91.69)	
	ST					
		Weekdays				<0.001
		≤2 hours/day	3368 (79.68)	1563 (77.22)	1805 (81.93)	
		>2 hours/day	859 (20.32)	461 (22.78)	398 (18.07)	
		Weekends				0.603
		≤2 hours/day	1739 (41.14)	841 (41.55)	898 (40.76)	
		>2 hours/day	2488 (58.86)	1183 (58.45)	1305 (59.24)	
		Whole week				0.960
		≤2 hours/day	1667 (39.44)	799 (39.48)	868 (39.40)	
		>2 hours/day	2560 (60.56)	1225 (60.52)	1335 (60.60)	
	Homework					
		Weekdays				0.700
		≤2 hours/day	2985 (70.62)	1435 (70.90)	1550 (70.36)	
		>2 hours/day	1242 (29.38)	589 (29.10)	653 (29.64)	
		Weekends				0.163
		≤2 hours/day	2621 (62.01)	1233 (60.92)	1388 (63.00)	
		>2 hours/day	1606 (37.99)	791 (39.08)	815 (37.00)	
		Whole week				0.463
		≤2 hours/day	2272 (53.75)	1076 (53.16)	1196 (54.29)	
		>2 hours/day	1955 (46.25)	948 (46.84)	1007 (45.71)	
PA and ST						<0.001
		PA- ST-	2308 (54.60)	1044 (51.58)	1264 (57.38)	
		PA- ST+	1410 (33.36)	667 (32.96)	743 (33.73)	
		PA+ ST-	252 (5.96)	181 (8.94)	71 (3.22)	
		PA+ ST+	257 (6.08)	132 (6.52)	125 (5.67)	
SRH, n (%)						<0.001
		Optimal	2821 (66.74)	1427 (70.50)	1394 (63.28)	
		Other	1406 (33.26)	597 (29.50)	809 (36.72)	

PA, physical activity; SB, sedentary behavior; SRH, self-rated health; MVPA, moderate-to-vigorous physical activity; ST, screen time; EPA, extracurricular physical activity.

- not meeting the recommendations.

+ meeting the recommendations.

Table 3 shows the association of PA with SRH. Boys and girls with at least 60 minutes per day MVPA on weekdays (boys: OR = 1.70, 95% CI = 1.35–2.13; girls: OR = 1.44, 95% CI = 1.14–1.83), weekends (boys: OR = 2.00, 95% CI = 1.59–2.50; girls: OR = 1.88, 95% CI = 1.46–2.41), and during a week (boys: OR = 1.92, 95% CI = 1.41–2.62; girls: OR = 3.40, 95% CI = 2.16–5.35) were more likely to report optimal health than those who did not achieve at least 60 minutes of MVPA per day. Boys with more than an hour a day of school-based PA were more likely to describe their health to be optimal (vs. ≤1 hour/day; OR = 1.49, 95% CI = 1.19–1.86), but not girls. Boys and girls who spent more than an hour a day in EPA were more likely to report optimal health (vs. ≤1 hour/day; boys: OR = 1.82, 95% CI = 1.42–2.34; girls: OR = 1.62, 95% CI = 1.23–2.13).

**Table 3:** ORs and 95% CIs for optimal self-rated health by physical activity (n = 4227).

Variables		Boys (n = 2024)		Girls (n = 2203)	
		OR	95% CI	OR	95% CI
MVPA					
	5d/wk vs. <5d/wk (weekdays)	**1.70**	1.35–2.13	**1.44**	1.14–1.83
	2d/wk vs. <2d/wk (weekends)	**2.00**	1.59–2.50	**1.88**	1.46–2.41
	7d/wk vs. <7d/wk (whole week)	**1.92**	1.41–2.62	**3.40**	2.16–5.35
School-based PA					
	>1h/d vs. ≤1h/d	**1.49**	1.19–1.86	1.08	0.86–1.34
EPA					
	>1h/d vs. ≤1h/d	**1.82**	1.42–2.34	**1.62**	1.23–2.13

OR, odds ratio; CI, confidence interval; PA, physical activity; MVPA, moderate-to-vigorous physical activity; EPA, extracurricular physical activity; d/wk, days/week; h/d, hours/day.

Adjusted by age and ethnicity.

Bold values are statistically significant.

Table 4 shows the association of SB with SRH. Boys with no more than two hours a day of SB on weekends were more prone to describe their health as optimal (vs. >2 hours/day; OR = 1.42, 95% CI = 1.02–1.99), but not girls. Girls with no more than two hours a day of SB during a week were more likely to report optimal SRH (vs. >2 hours/day; OR = 1.78, 95% CI = 1.19–2.68), but not boys. Girls with no more than two hours a day of ST on weekdays (vs. >2 hours/day; OR = 1.38, 95% CI = 1.10–1.74), weekends (vs. >2 hours/day; OR = 1.40, 95% CI = 1.14–1.71), and during a week (vs. >2 hours/day; OR = 1.42, 95% CI = 1.16–1.73) were more prone to report optimal health, but not boys. Boys and girls with no more than two hours a day on homework had not significantly higher OR for optimal SRH than those who spent more than two hours a day on homework.

**Table 4:** ORs and 95% CIs for optimal self-rated health by sedentary behavior (n = 4227).

Variables		Boys (n = 2024)		Girls (n = 2203)	
		OR	95% CI	OR	95% CI
SB					
	≤2h/d vs. >2h/d (weekdays)	0.91	0.74–1.12	1.04	0.86–1.26
	≤2h/d vs. >2h/d (weekends)	**1.42**	1.02–1.99	1.24	0.89–1.74
	≤2h/d vs. >2h/d (whole week)	1.25	0.86–1.82	**1.78**	1.19–2.68
ST					
	≤2h/d vs. >2h/d (weekdays)	0.98	0.78–1.24	**1.38**	1.10–1.74
	≤2h/d vs. >2h/d (weekends)	1.05	0.86–1.29	**1.40**	1.14–1.71
	≤2h/d vs. >2h/d (whole week)	1.06	0.86–1.29	**1.42**	1.16–1.73
Homework					
	≤2h/d vs. >2h/d (weekdays)	1.17	0.94–1.45	1.12	0.92–1.37
	≤2h/d vs. >2h/d (weekends)	1.05	0.86–1.29	1.10	0.91–1.33
	≤2h/d vs. >2h/d (whole week)	1.09	0.89–1.33	1.13	0.94–1.36

OR, odds ratio; CI, confidence interval; SB, sedentary behavior; ST, screen time; h/d, hours/day.

Adjusted by age and ethnicity.

Bold values are statistically significant.

Table 5 shows the combined associations of PA and SB with SRH. Meeting the PA and SB recommendations was significantly associated with optimal SRH in boys (OR = 1.61, 95% CI = 1.03–2.50) and girls (OR = 3.72, 95% CI = 2.08–6.65). Likewise, meeting the PA recommendations but not the SB recommendations was also significantly associated with optimal SRH among boys (OR = 2.40, 95% CI = 1.57–3.65) and girls (OR = 4.27, 95% CI = 2.09–8.75). However, meeting the SB recommendations but not the PA recommendations was significantly associated with optimal SRH in girls (OR = 1.39, 95% CI = 1.13–1.71) but not boys.

**Table 5:** Combined associations of physical activity and sedentary behavior with optimal self-rated health (n = 4227).

Variables	Boys (n = 2024)		Girls (n = 2203)	
	OR	95% CI	OR	95% CI
PA- ST-	1		1	
PA- ST+	1.12	0.90–1.39	**1.39**	1.13–1.71
PA+ ST-	**2.40**	1.57–3.65	**4.27**	2.09–8.75
PA+ ST+	**1.61**	1.03–2.50	**3.72**	2.08–6.65

OR, odds ratio; CI, confidence interval; PA, physical activity; SB, sedentary behavior.

- not meeting the recommendations.

+ meeting the recommendations.

Adjusted by age and ethnicity.

Bold values are statistically significant.

## 4. Discussion

This study is the first to explore the independent and joint associations of PA and SB with SRH in Chinese children and adolescents, contributing valuable insights to the existing, yet limited, body of literature. The findings showed that the majority of children and adolescents reported high levels of SRH, especially boys. This finding was in line with the HBSC survey results from the 40 countries [49, 50]. The study also shows that MVPA, school-based PA, and EPA were positively associated with optimal SRH. Conversely, SB and ST were negatively associated with optimal SRH. Meeting the PA and/or SB recommendations were beneficial to SRH, and these associations differed by gender and period.

In this study, children and adolescents who met PA guidelines recommendations were more likely to have optimal SRH. This result is consistent with previous studies investigating adolescents in European countries [33–36]. Most studies have confirmed that children and adolescents accumulating at least 60 min/day of MVPA can prevent disease and promote health [30, 51]. These findings suggest that engaging in MVPA for at least 60 minutes per day is not only beneficial to the objective health of children and adolescents, but also enhances their subjective perception of health. Moreover, children and adolescents with at least 60 min/day of MVPA on weekends were more likely to report optimal SRH than on weekdays. Given that school-aged children engaged in MVPA are mostly at school on weekdays and outside school on weekends. This result may imply that outside school MVPA is positively associated with optimal SRH, which is in line with the finding of Kantomaa et al. [52]. Previous research indicated that children and adolescents involved in activities outside of school exhibit higher self-esteem, reduced rates of depression, and are better positioned for optimal growth and development [53]. Outside school activities for children and adolescents are likely to revolve primarily around the family and community. Thus, these findings suggest strengthening MVPA interventions within family and community settings as a means to promote overall health in Chinese children and adolescents.

Our study uncovered a noteworthy distinction concerning the impact of school-based PA on SRH among boys and girls. Specifically, school-based PA exceeding one hour per day was found to be significantly associated with optimal SRH in boys but not in girls. A plausible explanation for this gender-based divergence may be the disparities in school-based MVPA levels between Chinese girls and boys, with previous research indicating that girls tend to engage in lower levels of school-based MVPA [54, 55]. Furthermore, it is worth noting that light PA does not exhibit significant associations with optimal SRH [27]. Therefore, it’s conceivable that, despite girls participating in school-based PA for more than one hour per day, they might allocate more time to light PA compared to boys. This suggests a critical need for the Chinese government to not only ensure that students engage in at least one hour of PA at school but also to emphasize the importance of maintaining PA at a moderate-to-vigorous intensity level. Additionally, our findings highlight the positive impact of EPA on SRH, as children and adolescents who participated in EPA for more than one hour daily reported optimal SRH levels. Extracurricular activities provide a unique avenue for children and adolescents to partake in PA, engage with peers, and cultivate friendships [56]. This result could be attributed to EPA’s role in establishing supportive social networks that contribute to the well-being of children and adolescents [56, 57]. Previous studies have demonstrated that adolescents who allocate more time to EPA tend to experience higher levels of life satisfaction and lower levels of anxiety and depression [58, 59], both of which have been associated with improved SRH among adolescents [16, 20]. These findings underscore the holistic benefits of EPA, not only in terms of physical well-being but also in nurturing positive mental health, ultimately contributing to better SRH among children and adolescents.

Beyond examining the impact of PA, our study delved into the relationships between SB and SRH in Chinese children and adolescents. Our study showed that girls who limited their ST were significantly associated with optimal SRH, whereas this correlation was not observed among boys. This outcome aligns with prior research findings [39]. A plausible explanation for this gender-based difference is that ST may exert a more pronounced negative influence on SRH in girls than in boys. Existing studies have pointed out that girls with extended periods of ST are more likely to experience poor mental health and be overweight than boys [60, 61], both of which have been demonstrated to have adverse effects on SRH among children and adolescents [16, 27]. These results are probably because girls are more prone to obesity and depression than boys in puberty [62, 63]. In addition, boys with more than two hours of SB per day on weekends were more likely to consider their health status as poor, but not girls. Considering that Chinese boys have shorter sleep duration than girls on weekends [64, 65]. A possible explanation is that boys spend more time playing video games than girls on weekends [66], which affects their sleep duration [67], and then indirectly leads to poor SRH status [24].

Doing homework for no more than two hours per day was not significantly associated with optimal SRH in both boys and girls, which reinforces the previous finding in Spain and Canada [27, 38]. Notably, the educational landscape in China is marked by intense academic pressure, stemming from a competitive education system, high parental expectations, and the pursuit of a successful future career trajectory [68, 69]. Therefore, one possible explanation for the observed trend is that students who dedicate more time to their homework tend to achieve better academic performance [70], which may reduce students’ academic pressure, in turn, the psychological discomfort caused by SB may be alleviated. To summarize, our study implies that, in the context of Chinese children and adolescents, reducing screen time appears to yield more tangible benefits for SRH than decreasing homework duration, this holds particularly true for girls.

In addition to the independent associations of PA and SB with SRH among Chinese children and adolescents, our study also delved into their combined effects. In our study, children and adolescents who met the PA recommendations were more likely to report optimal SRH regardless of whether they met the SB recommendations. In other words, these results suggest that meeting PA recommendations is more conducive to SRH in Chinese children and adolescents than the SB recommendations. This observation echoes a prior study, which demonstrated that increasing PA is more crucial than reducing sedentary time in enhancing SRH among Iranian children and adolescents [39]. Several factors may contribute to this outcome. First, not all types of sedentary behavior are inherently detrimental to health, which aligns with prior research indicating that the impact of a sedentary lifestyle can vary depending on the type of SB [38]. For example, brief and intermittent puzzle games can enhance mood, elicit positive emotions, promote relaxation, and reduce anxiety [71, 72]. Moreover, children and adolescents are in a stage of growth and development, and the health issues induced by SB may not have fully manifested, making them less aware of potential health problems. Furthermore, our study revealed that girls who met either the PA or SB recommendations, or both, exhibited higher odds ratios for reporting optimal SRH compared to boys. This finding indicated that achieving the PA and SB recommendations is more favorable for SRH in girls than boys. This finding holds particular importance, given that girls are generally more inclined to perceive their health as poor compared to boys [49]. Consequently, this result offers a potential avenue to narrow the gender-based SRH disparity by increasing compliance with PA and SB recommendations. Notably, there is substantial room for improvement in enhancing the compliance rate of these recommendations in China [47]. Therefore, it calls for the need to raise the compliance rate of PA and SB recommendations for enhancing the health of Chinese children and adolescents.

This study carries several limitations. First, data collection relied on self-report methods, which may introduce bias and discrepancies between reported results and objective realities. Nevertheless, self-report methods represent a common and practical approach in observational studies. Second, this research explored the associations of PA and SB with SRH, but it should be emphasized that the findings do not establish causal relationships between PA/SB and SRH in children and adolescents. In light of these constraints, future research could extend its scope by integrating objective measures for PA and SB and adopting longitudinal designs to delve deeper into these complex associations.

## 5. Conclusion

More PA was positively associated with optimal SRH in Chinese children and adolescents. A low level of ST is positively associated with optimal SRH in girls but not boys. Meeting the PA guidelines recommendations is more important than the SB guidelines recommendations for improving SRH in Chinese children and adolescents. Future studies can use objective PA and SB measures to explore the associations of PA and SB with SRH in China.

## Supporting information

S1 File (XLSX)
